# Supplementary material for: Reciprocal priming between receptor tyrosine kinases at recycling endosomes orchestrates cellular signalling outputs
Source: EMBO J. 2021 Jun 4;40(14):e107182. doi: 10.15252/embj.2020107182 (PMC8447605; doi:10.15252/embj.2020107182)
Supplement: Supplementary file 3 — Expanded View Figs PDF [file EMBJ-40-e107182-s004.pdf]

Expanded View Figures

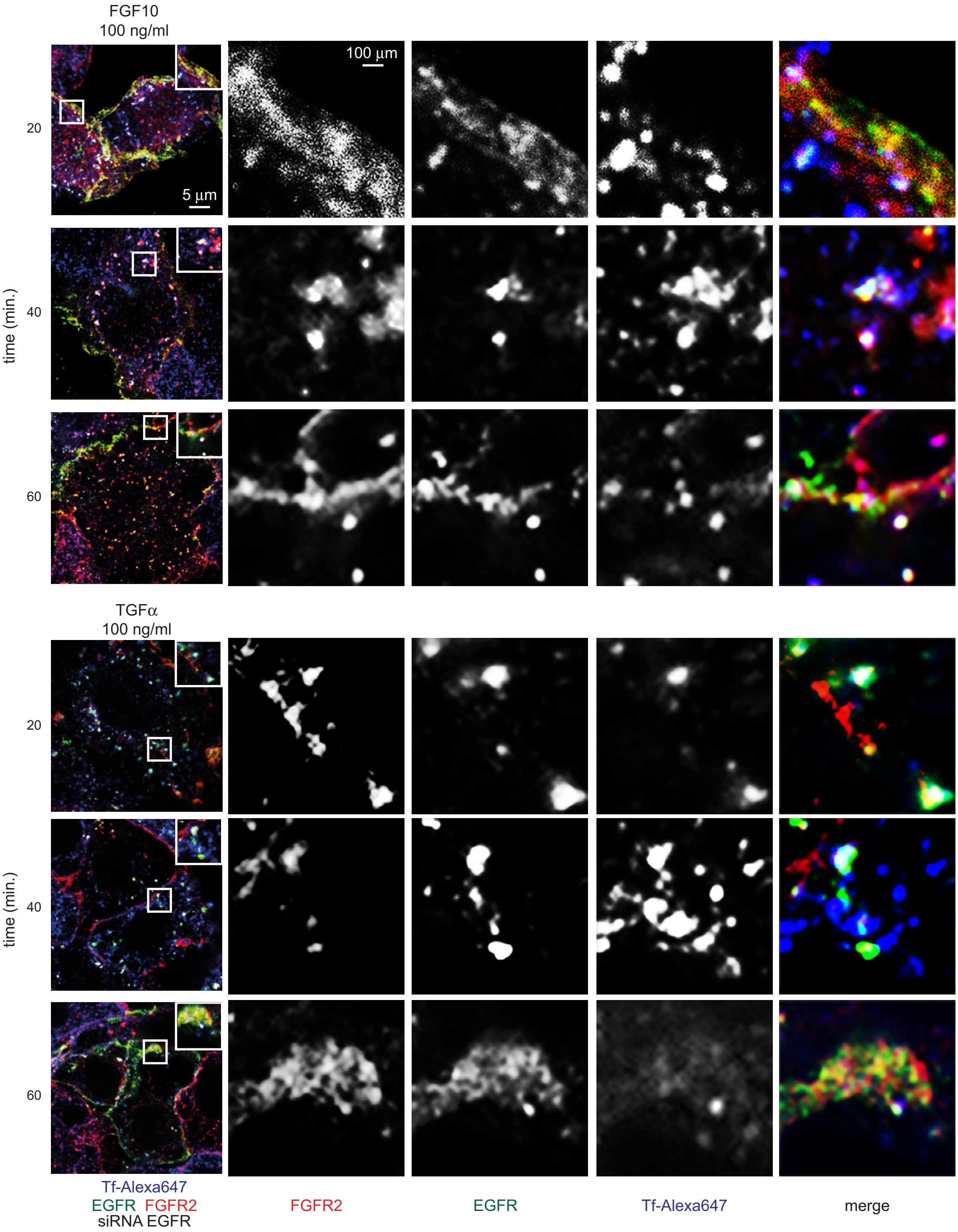

Fig EV1. Magnification of panels from stimulated T47D cells depleted of EGFR and transfection with wt EGFR.

Individual panels and merge of each magnified section of T47D depleted of EGFR by siRNA followed by transfection with wt EGFR and stimulated or not with either FGF10 or TGFα for the indicated time periods. Scale bars are indicated in the Fig. Panels Fig 5E are shown on the left for comparison.

© 2021 The Authors

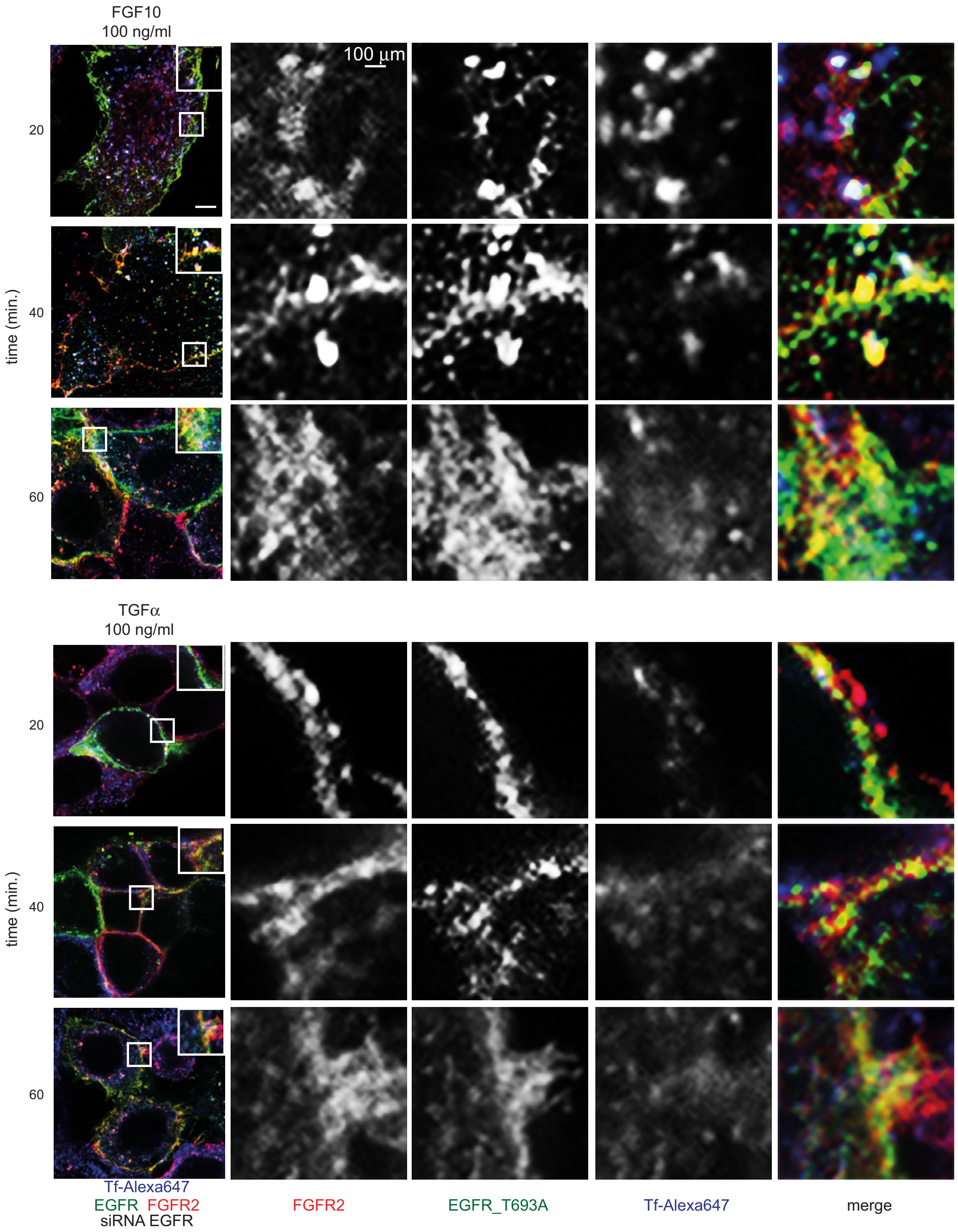

Fig EV2. Magnification of panels from stimulated T47D cells depleted of EGFR and transfection with the EGFR_T693A mutant.

Individual panels and merge of each magnified section of T47D depleted of EGFR by siRNA followed by transfection with EGFR_T693A and stimulated or not with either FGF10 or TGFα for the indicated time periods. Scale bars are indicated in the Fig. Panels from Fig 5E are shown on the left for comparison.

 © 2021 The Authors

Observation	Conditions	Figures
FGF7 doesn't cause recycling of FGFR2b	Confocal imaging, over expression of FGFR2b	Figure 1
FGF10 induces internalization and recycling of FGFR2b	Confocal imaging, membrane cytoplasm fractionation, Rab11 - APEX2 biotinylation, co-IP	Figures 1 and 5, Appendix Figures S4 and S6
Phosphorylation of EGFR at T693 downstream of FGFRs is found in recycling conditions	Three phosphoproteomics mass spec experiments (TPA1,2 and 3), Western Blots in several cell lines and organoid, over expression of FGFR2b.	Figures 2-5, Appendix Figures S2-5
Phosphorylation of EGFR at T693 relies on FGFR signalling through ERK	Inhibitor experiments	Figures 3-4
Phosphorylation of EGFR at T693 downstream of FGFRs requires active recycling	RCP/TTP depletion experiments, dominant negative dynamin experiments	Figure 2, Appendix Figure S4
Pre-treatment of FGF10 leads to enhanced ERK activity downstream of EGFR stimulation	Inhibitor experiments, western blots, qPCR of target genes, functional assays	Figure 4, Appendix Figure S5
FGF10 induced phosphorylation of T693 accumulates in the recycling endosomes after 20mins	Three phosphoproteomics mass spec experiments (TPA1,2 and 3), confocal imaging, co-IPs, Rab11 - APEX2 biotinylation experiment	Figures 2-5, Appendix Figures S4, S6-7
EGFR_T693A that can't internalize leads to faster FGFR2 trafficking back to PM	Confocal imaging, co-IPs	Figure 5, Appendix Figure S6
EGFR_T693A that can't internalize alters FGFR2 signalling and cellular outputs	Phosphoproteomics, western blots, functional assays	Figures 5-7, Appendix Figures S6-7

Fig EV3. Table summarizing the main findings of this study.

Summary table of key observations made in this study, a brief description of the conditions these observations were made under, and the location of key results underpinning them.

© 2021 The Authors
